# Intrinsic signal optoretinography of dark adaptation abnormality due to rod photoreceptor degeneration

**DOI:** 10.3389/ebm.2024.10024

**Published:** 2024-01-31

**Authors:** Jie Ding, Tae-Hoon Kim, Guangying Ma, Xincheng Yao

**Affiliations:** ^1^ Department of Biomedical Engineering, University of Illinois Chicago, Chicago, IL, United States; ^2^ Department of Ophthalmology and Visual Sciences, University of Illinois Chicago, Chicago, IL, United States

**Keywords:** rd10, rod degeneration, OCT, dark adaptation, IOS

## Abstract

This research aims to investigate the potential of using intrinsic optical signal (IOS) optoretinography (ORG) to objectively detect dark adaptation (DA) abnormalities related to rod photoreceptor degeneration. Functional optical coherence tomography (OCT) was employed in both wild-type (WT) and retinal degeneration 10 (rd10) mice to conduct this assessment. Dynamic OCT measurements captured the changes in retinal thickness and reflectance from light-to-dark transition. Comparative analysis revealed significant IOS alterations within the outer retina. Specifically, a reduction in thickness from external limiting membrane (ELM) peak to retinal pigment epithelium (RPE) peak was observed (WT: 1.13 ± 0.69 µm, 30 min DA; rd10: 2.64 ± 0.86 µm, 30 min DA), as well as a decrease in the intensity of the inner segment ellipsoid zone (EZ) in 30 min DA compared to light adaptation (LA). The reduction of relative EZ intensity was notable in rd10 after 5 min DA and in WT after 15 min DA, with a distinguishable difference between rd10 and WT after 10 min DA. Furthermore, our findings indicated a significant decrease in the relative intensity of the hypo-reflective band between EZ and RPE in rd10 retinas during DA, which primarily corresponds to the outer segment (OS) region. In conclusion, the observed DA-IOS abnormalities, including changes in ELM-RPE thickness, EZ, and OS intensity, hold promise as differentiators between WT and rd10 mice before noticeable morphological abnormalities occur. These findings suggest the potential of this non-invasive imaging technique for the early detection of dysfunction in retinal photoreceptors.

## Impact statement

Various eye diseases can lead to photoreceptor dysfunction, with rod photoreceptors being particularly susceptible. Therefore, it is crucial to assess rod photoreceptor function early for detecting eye diseases. This study showcases the potential of using intrinsic signal optoretinography to objectively detect dark adaptation abnormalities associated with rod photoreceptor degeneration.

## Introduction

Ocular conditions like age-related macular degeneration (AMD) [1–3], retinitis pigmentosa (RP) [4, 5], and diabetic retinopathy (DR) [6, 7], can result in photoreceptor dysfunctions and eventually vision loss. Numerous eye diseases primarily affect retinal photoreceptors. Presently, there is no definitive cure for these degenerative retinal diseases, which cause permanent damage to photoreceptors and the retinal pigment epithelium (RPE). To prevent vision loss from retinal degeneration, preserving function and identifying progression early are crucial. Therefore, detecting potential biomarkers promptly is essential in prevention of these retinal diseases [8, 9].

Although structural changes like drusen and macular pigmentary abnormalities are valuable for evaluating eye conditions in AMD patients, they do not offer a comprehensive assessment of retinal function [10]. Physiological abnormalities in retinal cells can manifest before any noticeable morphological changes, such as cell loss or thickness alterations. Hence, it is crucial to evaluate the physiological conditions of the retina for the early detection of eye diseases.

Electrophysiological methods like electroretinography (ERG) objectively assess retinal physiology [11, 12]. It is known that ERG a-wave reflects stimulus-evoked physiological activation of photoreceptors, and b-wave reflects the physiological dynamics of neurons. ERG abnormalities were reported in eye diseases [13] and neurodegenerative conditions [14, 15] at an early stage. However, it can be complicated to accurately interpret ERG alterations in diseased conditions since its signal represents combined activities of retinal photoreceptors and inner neurons. It’s reported that a-wave abnormality may be attributable to a contamination of post-photoreceptor abnormality in retinal dysfunction [16]. Moreover, the spatial resolution of ERG is relatively low, and thus it is difficult to correlate physiological changes to morphological abnormalities revealed in high resolution imaging.

Intrinsic optical signal (IOS) imaging [17–22], also known as optophysiology [23], optoretinogram [24–28] or optoretinography (ORG) [29–33] allows for simultaneous assessment of retinal morphology and physiology. Optical coherence tomography (OCT) provides non-invasive depth-resolved imaging of retinal layers, making OCT-based intrinsic signal ORG a promising approach for studying human and animal retinas [27–31].

Functional OCT has been utilized to monitor photoreceptor-IOS changes in retinal degeneration 10 (rd10) mice over time [32]. Rd10 mice are a recognized model of rod photoreceptor degeneration [34–38] due to a spontaneous mutation in the genes encoding β-subunit of rod-phosphodiesterase (PDE), resulting in cGMP accumulation and subsequent rod degeneration [37–42]. Since the rods can express approximately 40% of endogenous PDE [41, 43], this degeneration in rd10 mice occurs relatively slowly, starting around postnatal day 16 (P16) to P17. After P21, the outer retina is massively reduced and unable to distinguish. Photoreceptor-IOS abnormalities were previously detected in rd10 mice at P17 by using functional OCT [32]. With a rapid time course to follow visible light stimulus, fast photoreceptor-IOS invoked by the light stimulation can reflect the early phase of phototransduction reaction chains [44]. Photoreceptor-IOS abnormalities were previously detected in rd10 mice at P17 using functional OCT. Fast photoreceptor-IOS responses were found to be similar in both wild-type (WT) and rd10 mice at P14, suggesting that this response occurs before PDE activation in phototransduction reaction chains [41]. The relative expression levels of rhodopsin and transducin in rd10 mice are comparable to those in WT mice [41, 43, 45], indicating that fast photoreceptor-IOS alone is not enough to evaluate PDE deficiency in rd10 before P15.

Dark adaptation (DA) is a method used to assess functional changes in retinal photoreceptors. During DA, eyes are exposed to dark environment and the retina shifts from cone to rod activities in the absence of light. PDE activation can impact the metabolic function of retinal photoreceptors, and thus may cause DA abnormalities. ERG studies have suggested that DA abnormalities may reflect retinal metabolic issues [40], as an early indicators of rod photoreceptor degeneration [46, 47]. The photoreceptor inner segment ellipsoid zone (EZ) is known to be the metabolic center with abundant mitochondria, and it has been demonstrated as a non-invasive OCT marker that reflects optical index changes of cell substances due to photoreceptor activity [48–50]. Recent research demonstrated the monitoring of DA-IOS kinetics within the outer retina, with a focus on the dynamics of the ellipsoid zone (EZ), in healthy adult mice [51]. This study aims to determine if DA-IOS kinetics in the outer retina, which involves downstream transducin activation and photoreceptor metabolism, can detect PDE deficiency-induced photoreceptor abnormalities at P14.

## Materials and methods

### Animal preparation

OCT images were collected from retinas of Six C57BL/6J mice and nine B6.CXB1-Pde6brd10/J mice (Jackson Laboratory, Bar Harbor, Maine, United States). Pre-experiment, the mouse eyes underwent light adaptation in a well-lit room for more than 5 h [52]. A mixed solution of 100 mg/kg ketamine and 5 mg/kg xylazine was used for anesthesia by intraperitoneal injection. Following anesthesia, 0.5% tropicamide was used for eye dilation. GenTeal gel (Alcon Laboratories, Fort Worth, Texas, United States) was applied to mice eyes during the OCT imaging to prevent dryness of the cornea. Post-experiment, mice were kept on a heating pad before recovering from anesthesia and then caged in the animal facility in the Biological Resources Laboratory at the University of Illinois Chicago.

### OCT system

A custom-designed OCT system described in a previous paper [51] was used for imaging. Light source was a superluminescent diode (SLD) with a wavelength of 810 nm and a bandwidth of 100 nm (D-810-HP, Superlum, Carrigtwohill, County Cork, Ireland). Light from the SLD was split into the subject and reference arms using a fiber coupler (75:25, TW850R5A2, Thorlabs, Newton, New Jersey, United States). For the spectrometer, a custom-designed setup was assembled, featuring a line CCD camera with 2048 pixels (OCTOPLUS, e2v, Chelmsford, United Kingdom) and a transmission grating with 1200 lines per millimeter (Wasatch Photonics, West Logan, Utah, United States). The sensitivity of the OCT system was assessed using a mirror as the imaging subject, yielding a signal-to-noise ratio of 40 dB. The axial resolution was approximately 2.1 μm and lateral resolution was approximately 11 μm in mouse eye.

### Data acquisition

OCT volumes were acquired in a well-lit laboratory room at noon to reduce the impact of circadian rhythm-related disk shedding [52, 53]. The mouse was fully anesthetized and transferred to an adjustable mount. The mouse head was securely fixed by a bite bar and two ear bars to prevent physical movement which may cause the imaging location or focus point change. During OCT imaging, a rodent surgical heating pad was used to keep the body warm.

**FIGURE 1:**
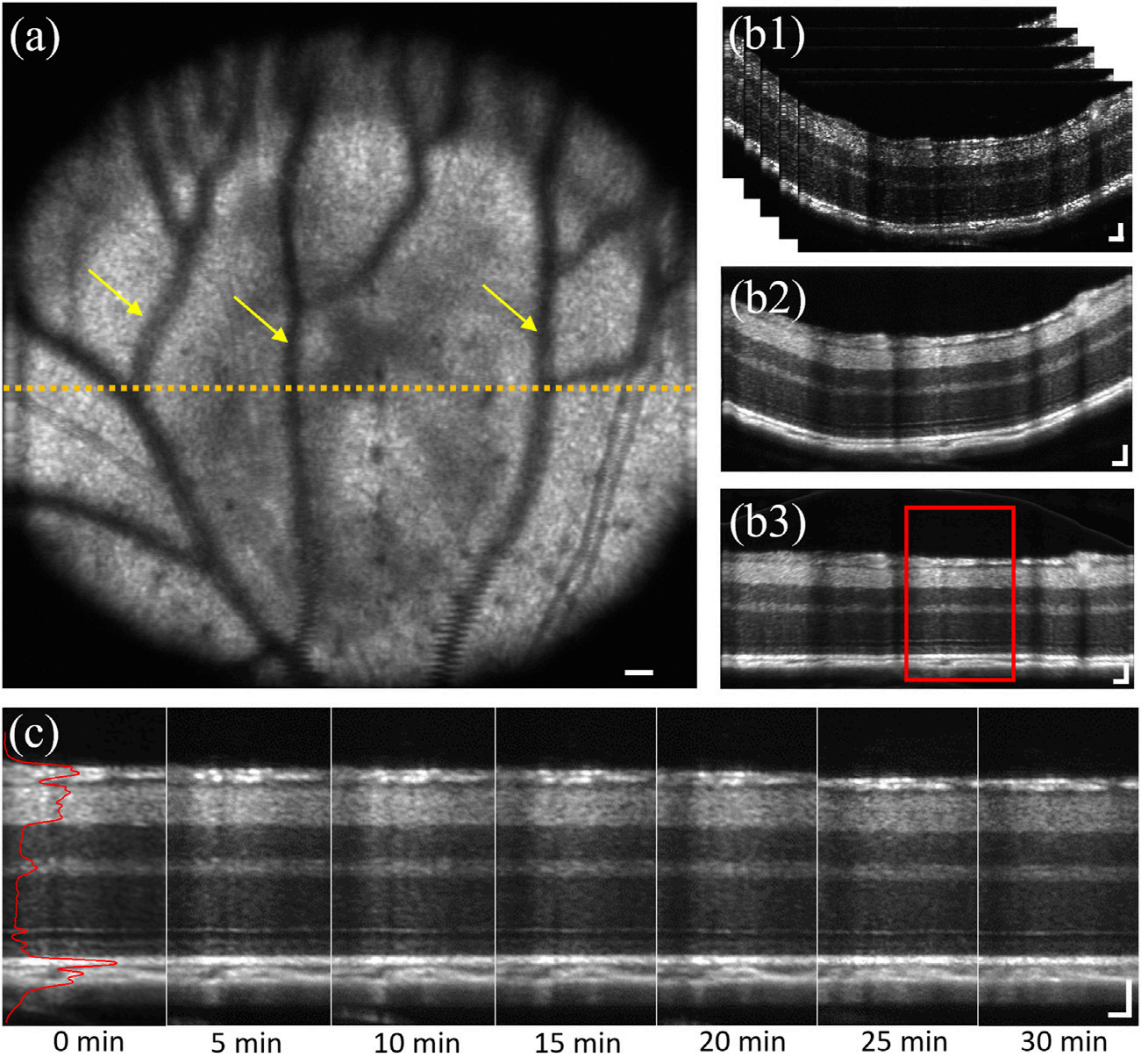
**(A)** Representative en face image of the dorsal quadrant of a mouse retina. Yellow arrows indicate projection shadows of hyaloid vessels. Yellow dashed line represented the selected area, approximately 0.7 mm from optical nerve head. **(B1)** Representative image stack of selected 50 B-scans with 4 repetitions, corresponding to the retinal region marked by yellow dashed line in a. **(B2)** Representative averaged OCT B-scan of b1. **(B3)** Flattened illustration of **(B2)**. **(C)** Representative sequential recording, corresponding to the red window in **(B3)**. Solid red line represents the averaged A-line reflectance profile of the B-scan at 0 min DA. Scale bar: 50 µm.

Mouse retinas were measured at the dorsal quadrant over a 1.4 mm*1.4 mm area (Figure 1A). Each mouse retina was imaged for one experiment that contained 7 sequential OCT volumes for a total of 30 min recording, with a 5 min interval. The first OCT volume was imaged and adjusted under normal lighting conditions, while the subsequent six OCT volumes were sequentially recorded under dark conditions using identical settings. The focus point and location of the imaging area were optimized before turning off lights. Every OCT volume comprised 500 B-scans with 4 repeated scannings, covering a 1.4 mm × 1.4 mm area. Each B-scan contained 500 A-lines. The fps was 80 B-scans per second. All OCT operations were completed by the same person to reduce errors.

### Image processing

Reconstruction of OCT volumes was processed through MATLAB using a custom-designed algorithm including k-sampling, interferogram extraction, apodization, fast Fourier transform (FFT). After OCT reconstruction, 50 consecutive B-scans with 4 repetitions at the retinal cross-section approximately 0.7 mm away from the optical nerve head (ONH) were selected for stack registration (yellow dash line in Figure 1A). The image stack contained 200 B-scans in total (Figure 1B1). Then the stack was registered using the first B-scan as a reference and subsequently aligned the following 199 B-scans to remove stack movement caused by mouse breathing. After that, the image stack was averaged into one B-scan (Figure 1B2) to increase image fidelity and reduce sparkle difference. Then, the A-lines of the B-scan were realigned for image flattening (Figure 1B3), which used the middle one A-line as a reference and all adjacent A-lines were aligned with a subpixel registration algorithm. Next, a central portion of the flattened B-scan, covering 100 to 150 A-lines, was extracted to generate an averaged A-line reflectance profile (Figure 1C). The reason to choose center A-lines instead of the whole B-scan was to avoid the shadow area caused by hyaloid vessels [54] (yellow arrows, Figure 1A) and distortion caused by the retina angle at both edges. Also, large blood vessels were avoided when choosing center A-lines, because the retina was thickened where the large blood vessel walls embedded. Retinal thickness and layer intensity were measured based on the A-line profile normalized using the outer nuclear layer (ONL) intensity as a reference [55–57].

Statistical analysis was performed using Origin’s one-way repeated-measures analysis of variance (ANOVA) with Bonferroni correction. A significance level of *p*-values < 0.05 was used, and standard deviation was represented as the error bar for each data point.

## Results

### Morphological changes caused by photoreceptor degeneration

**FIGURE 2:**
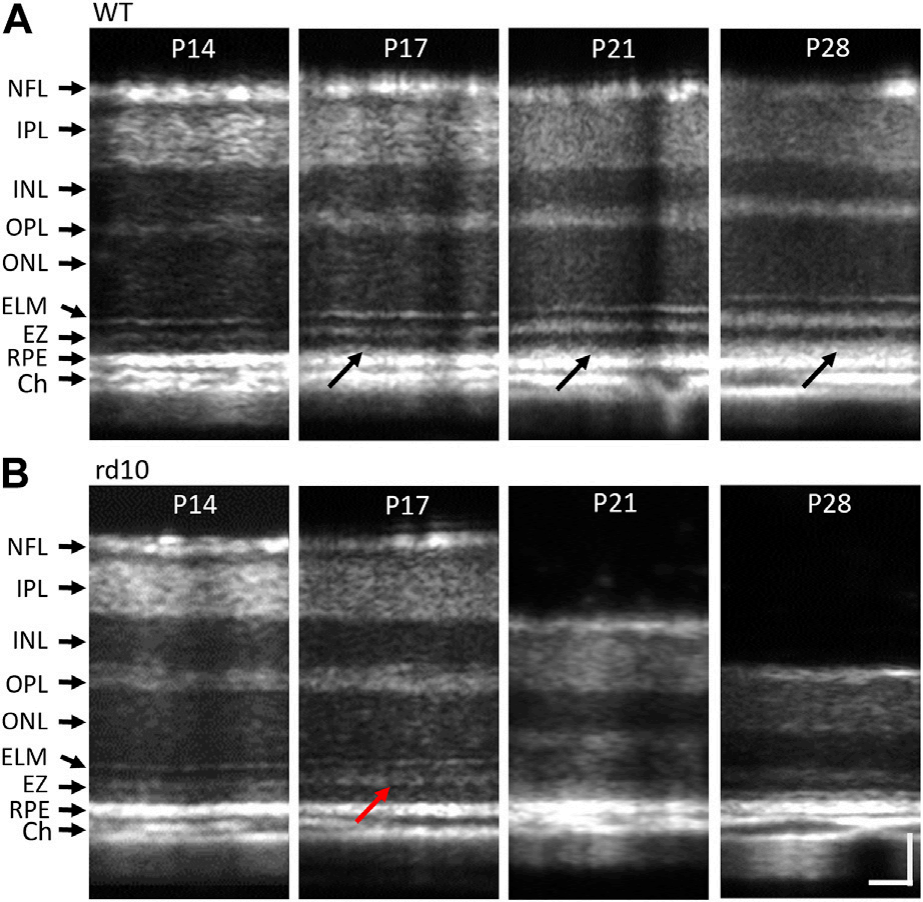
Representative morphological OCT images of WT **(A)** and rd10 **(B)** retinas at P14 to P28. The red arrow in figure 2b2 marks the OS region of rd10 started dissembling at P17. NFL, nerve fiber layer; IPL, inner plexiform layer; INL, inner nuclear layer; OPL, outer plexiform layer; ONL, outer nuclear layer; ELM, external limiting membrane; EZ, inner segment ellipsoid; RPE, retinal pigment epithelium; Ch, choroid. Scale bar: 50 µm.

Morphological comparison was conducted to investigate the structural changes caused by photoreceptor degeneration. Representative OCT images from WT and rd10 mice at P14, P17, P21, and P28 under light-adapted (LA) conditions were presented in Figure 2. Retinal OCT morphological changes were observed in rd10, compared to WT mice from P17. Specifically, rd10 retinas exhibited unclear external limiting membrane (ELM) and “swollen” EZ structures (red arrow, Figure 2B), with significant reduction in outer retinal thickness at P21 and P28. Also, the interdigitation zone (IZ) could be observed in WT retinas from P17 to P28 (dark arrows, Figure 2A), whilst it’s not shown in rd10 retinas. As degeneration signs began around P17, morphologically intact retinas at P14 were chosen for comparative OCT measurements and analysis in the following sections. This stage represents an early phase before significant abnormality appeared.

### Functional changes of retinas during light to dark transition

**FIGURE 3:**
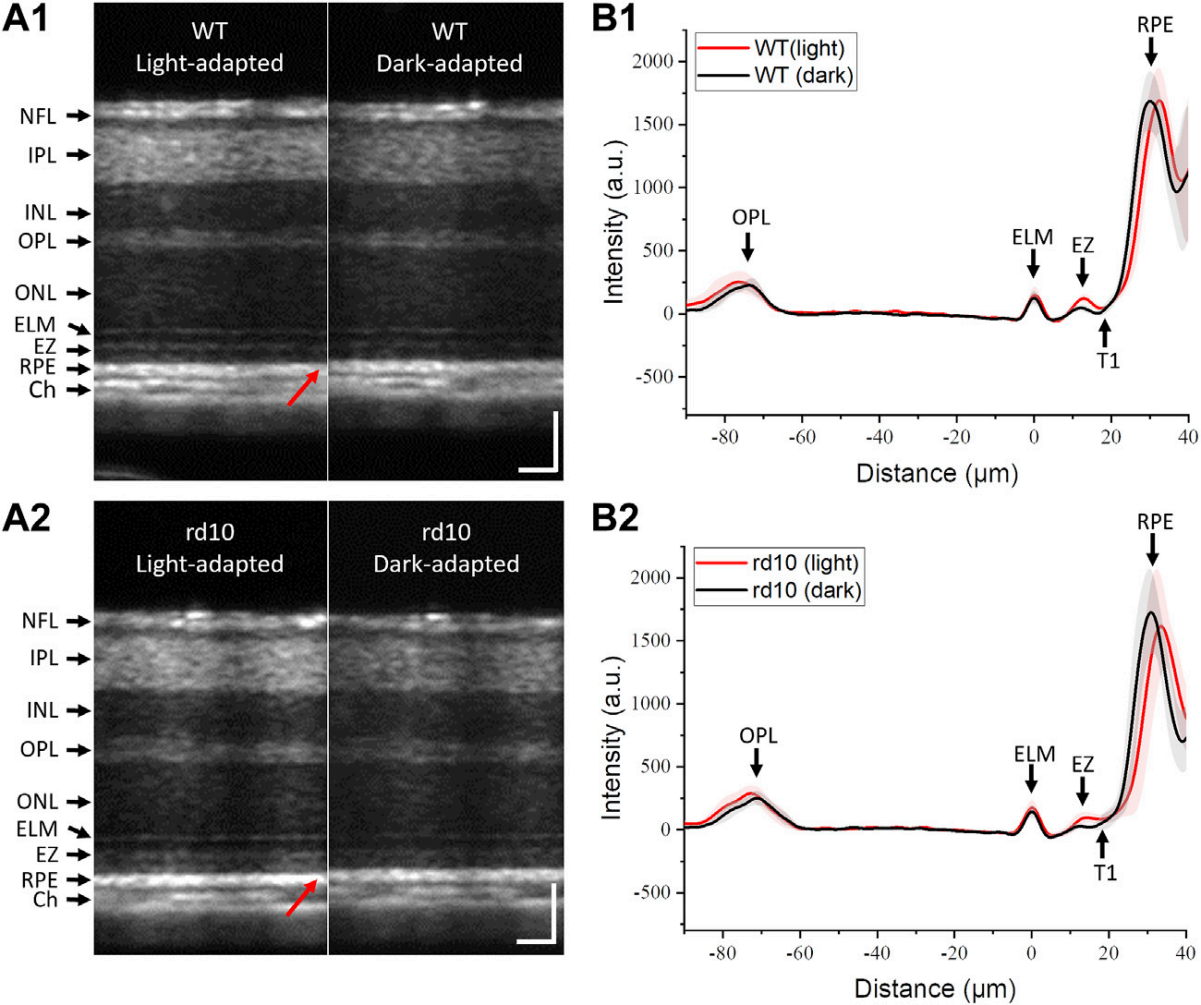
Representative OCT images of mouse retinas from WT **(A1)** and rd10 **(A2)** in LA and after 30 min DA; Averaged A-line profiles of OCT images from six WT **(B1)** and nine rd10 mice **(B2)** in LA and after 30 min DA. Standard deviations were represented as shadows adjacent to the lines. NFL: nerve fiber layer; IPL: inner plexiform layer; INL: inner nuclear layer; OPL: outer plexiform layer; ONL: outer nuclear layer; ELM: external limiting membrane; EZ: inner segment ellipsoid; RPE: retinal pigment epithelium; Ch: choroid; T1: trough 1. Scale bar: 50 µm.

OCT analysis of P14 WT and rd10 retinas under LA and DA conditions were compared in Figure 3. Inner retinal morphology showed no obvious differences in WT (Figure 3A1) and rd10 (Figure 3A2) mice between LA and DA. In both groups, the thickness from ELM peak to RPE peak (highlighted with red arrows, Figure 3A) was reduced during DA compared to LA. Figure 3B showed averaged A-line profiles of retinas form six WT mice and nine rd10 mice at LA condition and after 30 min of DA to further characterize the observed retinal shrinkage in WT (Figure 3B1) and rd10 (Figure 3B2) retinas. All mouse retinas were measured at P14. Standard deviations were represented as shadows accompanying the lines. For easy comparison, the location of ELM peak was normalized to 0 on the X-axis. In both groups, the RPE peak shifted toward the ELM in DA compared to LA. Additionally, OCT band intensities were altered, with a notable decrease in EZ peak intensity in both WT and rd10 during DA compared to LA. The OPL and ELM peak values also slightly decreased in DA.

A clear hyporeflective band, T1, situated between EZ and RPE, was visible in WT retinas (Figure 3B1). However, T1 was elusive in rd10 retinas after 30 min DA (Figure 3B2). As the T1 primarily corresponds to the photoreceptor outer segment (OS) region due to its location between EZ and RPE, this observation might indicate potential photoreceptor OS abnormalities, suggesting a physiological defect in rd10 photoreceptors.

### Comparative analysis of retinal changes during light to dark transition

We proceeded to compare DA-IOS kinetics in WT and rd10 retinas during a 30 min transition from light to dark. Time-lapse OCT images were recorded, including seven sequential OCT volumes from 0 (LA) to 30 min of DA at a 5-min interval.

**FIGURE 4:**
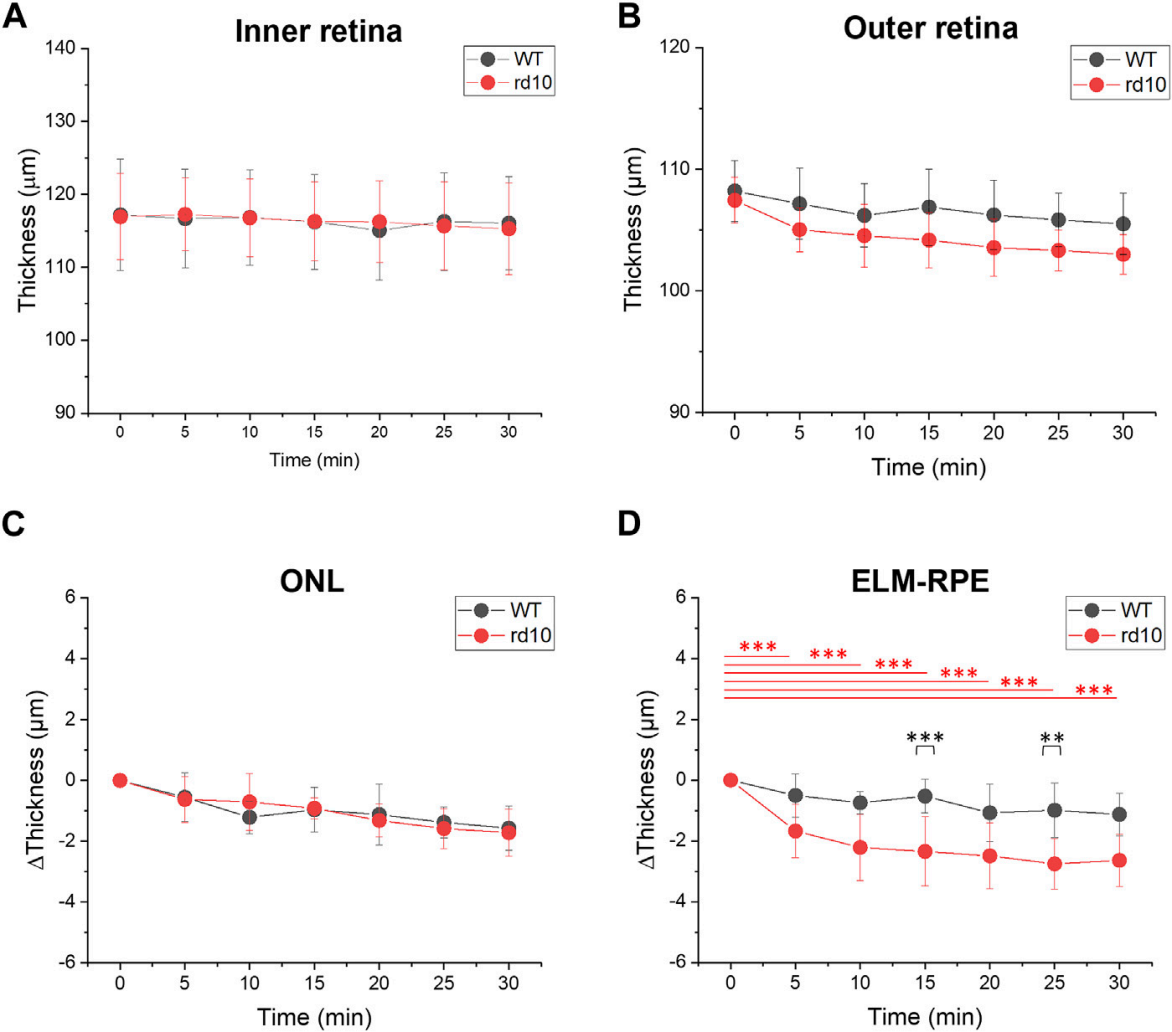
Comparative analysis of outer retina thickness changes during light to dark transition. **(A)** Inner retina (NFL-OPL) thickness; **(B)** Outer retina (OPL to RPE) thickness; **(C)** ONL (OPL to ELM) thickness change; **(D)** ELM to RPE thickness change. Standard deviation was represented as the error bar for each data point. **p < 0.001, ***p < 0.001, compared to 0 min within group and at same time point between groups, using one-way ANOVA with the Bonferroni correction. Data was averaged from six WT and nine rd10 retinas.

The thickness changes in inner and outer retina during DA were quantitatively assessed. Figure 4A illustrated inner retina thickness changes in both groups, with no significant alterations observed in the inner retina (NFL-OPL). Conversely, outer retina thickness (OPL-RPE) gradually decreased during DA, aligning with the shrinkage of outer retina observed in DA conditions in Figure 3. Figures 4C, D separately illustrated the ONL (OPL-ELM) and ELM-RPE thickness reduction. The thickness at LA condition (0 min) was set as standard and subtracted by the thicknesses at following time points. Both ONL and ELM-RPE thicknesses consistently decreased during DA. Results were shown in Figures 4C, D, that the ONL thickness change of WT (1.57 ± 0.73 µm, 30 min DA) was similar with rd10 (1.72 ± 0.78 µm, 30 min DA), however the ELM-RPE thickness change of WT (1.13 ± 0.69 µm, 30 min DA) was smaller than the rd10 (2.64 ± 0.86 µm, 30 min DA). Statistical significance was shown in rd10 after 5 min DA but not in WT (*p* < 0.001). Additionally, at time point 15 and 25, the difference was significant between the two groups (*p* < 0.01).

**FIGURE 5:**
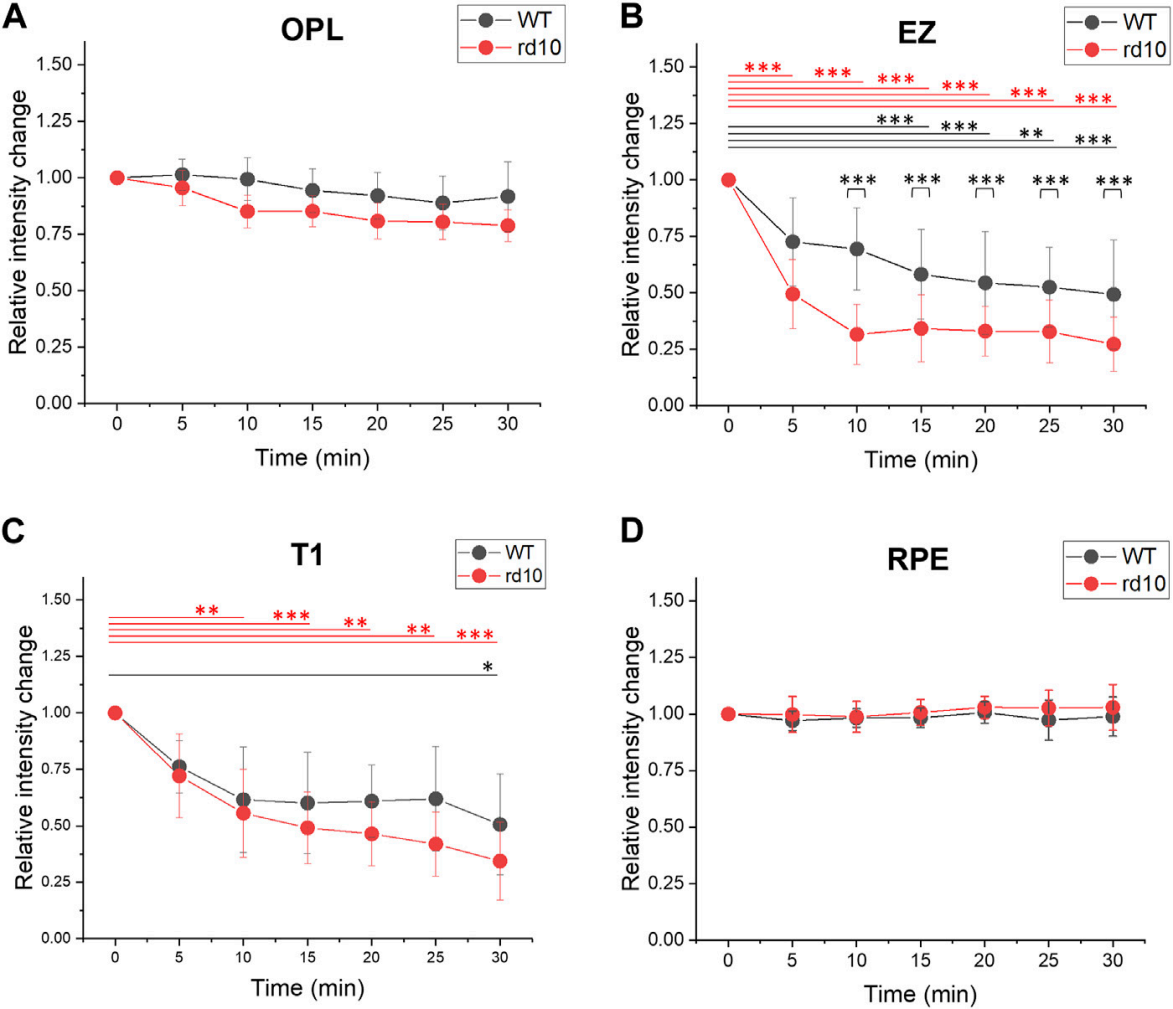
Comparative analysis of outer retina relative intensity changes during light to dark transition. **(A)** OPL relative intensity change; **(B)** EZ relative intensity change; **(C)** T1 relative intensity change. **(D)** RPE intensity change. Standard deviation was represented as the error bar for each data point. *p < 0.05, **p < 0.01, ***p < 0.001, compared to 0 min within one group and at same time point between two groups, using one-way ANOVA with the Bonferroni correction. Data was averaged from six WT and nine rd10 retinas.

Figure 3B demonstrated a decrease in OCT intensity in the outer retina, particularly the EZ band in both retinas. Figure 5 followed this finding to further compare the relative intensity changes from outer retinal layers. The results indicated similar relative intensity changes in OPL (Figure 5A) and RPE (Figure 5D) bands between WT and rd10. However, significant DA-IOS kinetic differences were observed in EZ and T1 (Figures 5B, C). A significant intensity reduction was observed in EZ after 5 min DA in rd10 retinas (*p* < 0.001), while it occurred after 15 min DA in WT retinas (*p* < 0.001). Also, statistical difference was shown between WT and rd10 after 10 min DA (*p* < 0.001). Regarding the relative intensity reduction of T1, Figure 5C demonstrated the notable decrease in rd10 after 10 min of DA (*p* < 0.01), while in WT, this decrease was observed only after 30 min of DA (*p* < 0.05). Together, these results indicated that during DA the intensity of EZ peak and OS region decreased rapidly in rd10, compared to WT.

## Discussion

Functional OCT was conducted to characterize morphological measurements and DA-IOS kinetics at the outer retina in WT and rd10 mice. First, we observed unclear ELM and “swollen” EZ at P17, as well as reduced outer retina at P21 in rd10 OCT images, and the IZ was observed only in WT OCT images after P17. According to these morphological changes, we then studied structural intact retinas at P14 using dark adaptation method, to observe functional morphological changes. Both WT and rd10 retinas displayed a decrease in ELM-RPE thickness and a reduction in EZ intensity during DA, as shown in Figures 4, 5. The observation aligns with prior reports of outer retina shrinkage in dark conditions [51, 52, 58]. However, it’s worth noting that the transient thickness change of ELM-RPE in rd10 during DA was larger compared to WT, as indicated in Figure 4D. The EZ and T1 intensity changes were distinguishable quickly in rd10 after 5 min and 10 min DA (Figures 5B, D). These results suggested that DA-IOS kinetics, particularly the ELM-RPE thickness, EZ and OS body intensity, can provide sensitive biomarkers for functional assessment of photoreceptor physiology.

Compared to previous study [51], the DA changes in thickness and intensity were more significant and the OCT reflectance itself was brighter in mature WT retina. A potential explanation could be that the metabolic condition and response in adult mouse retina are known to become more robust with maturation which was reflected in ERG [59]. In addition, it has been reported that at the structural and molecular level, photoreceptors are fully functional by P30, and protein expressions as well as synapse activity become mature after P30 [60].

We speculated some potential mechanisms behind the DA-IOS changes, specifically the observed shrinkage of ELM-RPE thickness and reduction of EZ intensity from P14 retinas. Many studies reported that enhanced RPE pumping activity [40, 58, 61, 62] and mitochondrial morphological changes during DA [50, 61] may reduce the fluid content, which leads to shrinkage of ELM-RPE thickness and decrease of OCT reflectance. Higher oxidative stress in rod photoreceptors of rd10 [63] could cause oxidative damage to photoreceptors and affect their functions [64]. Increased oxygen supply to the photoreceptor increased the respiratory metabolites including water content produced by mitochondria [65, 66]. After DA, the accumulation of metabolic fluid between ELM-RPE is reduced and leads to thickness shrinkage in the rd10 retina [58]. Thus, we hypothesized that the enhanced water content removal together with mitochondrial activity contributes to accelerated shrinkage of ELM-RPE thickness and reduction of EZ intensity in rd10.

Decrease of internal osmotic pressure in DA resulting in reversed cytoplasmic swelling and less backscattering could be another potential mechanism behind these DA-IOS changes. In the presence of light, rhodopsin absorbs photons and becomes active, then activates transducin and subsequent PDE-mediated cGMP hydrolysis, which turns off ion channels. This process may increase osmotic pressure with extra osmolytes and result in cytoplasmic swelling, causing increased ELM-RPE thickness and EZ intensity [67, 68]. However, in DA without light, rhodopsin is inactivated, and the cascade gradually stops. Without transducin activating PDE, cGMP contributes to ion flux through open ion channels, reducing internal osmotic pressure and promoting cytoplasmic fluid removal, leading to shrinkage of ELM-RPE thickness and reduction of EZ intensity [67]. This process occurs after transducin activation, which was expressed comparably in WT and rd10 retinas at P14 [41]. Therefore, in both groups the thickness shrinkage and intensity reduction were observed. However, rd10 retinas at P14 exhibited accelerated reductions in ELM-RPE thickness and EZ intensity compared to WT, possibly due to the pre-existing PDE deficiency in rd10 mice present from birth [38, 41, 43, 45], leading to less functional PDE proteins and higher cGMP accumulation in the OS [37]. The increased cGMP concentration may result in faster osmotic pressure reduction with restoration of cytoplasmic fluid removal in DA, contributing to accelerated thickness shrinkage and reduced backscattering in retinal photoreceptors of rd10 mice.

Based on the A-line reflectance profiles, we did not observe a hyperreflective peak (IZ) near RPE in P14 mouse like in adult WT mouse retina [51], but only the hyporeflective band T1 between EZ and RPE. Interestingly, the T1 intensity in rd10 was higher than that in WT in light condition, and it was almost same as the EZ intensity after dark adaptation (Figure 3B). It is known that hyporeflective band T1 between EZ and RPE was most correlated with OS body [32]. The intensity difference in OS may be result from the deficient PDE level in rd10 retina at P14 and need further investigation. Together, the observation of T1 intensity suggested that possible OS abnormality occurred during DA in rd10 retinas at P14.

## Conclusion

Comparative OCT revealed DA-IOS markers, such as shrinkage of ELM-RPE thickness and reduction of EZ and T1 relative intensities, to differentiate WT and rd10 retinas at around P14 before notable morphological abnormality. Both WT and rd10 retinas consistently showed ELM-RPE thickness shrinkage together with EZ intensity reduction after DA. The DA caused thickness and intensity responses were observed to be significantly larger and quicker in rd10 compared to WT, providing non-invasive IOS markers for early detection of photoreceptors dysfunction due to degeneration. The hyporeflective band T1, corresponding to photoreceptor OS, also showed obvious intensity difference. The assessment of DA-IOS kinetics through intrinsic signal ORG presents a valuable non-invasive approach to detect early signs of rod photoreceptor degeneration.

## Data Availability

The raw data supporting the conclusion of this article will be made available by the authors, without undue reservation.

## References

[1] Curcio CA Medeiros NE Millican CL. Photoreceptor loss in age-related macular degeneration. Invest Ophthalmol Vis Sci (1996) 37:1236–49. 8641827

[2] Yang S Zuo C Xiao H Mi L Luo G Xu X Photoreceptor dysfunction in early and intermediate age-related macular degeneration assessed with mfERG and spectral domain OCT. Doc Ophthalmol (2016) 132:17–26. 10.1007/s10633-016-9523-4 26754967

[3] Jackson GR Owsley C Curcio CA. Photoreceptor degeneration and dysfunction in aging and age-related maculopathy. Ageing Res Rev (2002) 1:381–96. 10.1016/s1568-1637(02)00007-7 12067593

[4] Holopigian K Seiple W Greenstein VC Hood DC Carr RE. Local cone and rod system function in patients with retinitis pigmentosa. Invest Ophthalmol Vis Sci (2001) 42:779–88. 11222541

[5] Wen Y Locke KG Hood DC Birch DG. Rod photoreceptor temporal properties in retinitis pigmentosa. Exp Eye Res (2011) 92:202–8. 10.1016/j.exer.2010.12.014 21219898 PMC3057282

[6] Holopigian K Greenstein VC Seiple W Hood DC Carr RE. Evidence for photoreceptor changes in patients with diabetic retinopathy. Invest Ophthalmol Vis Sci (1997) 38:2355–65. 9344359

[7] McAnany JJ Park JC. Cone photoreceptor dysfunction in early-stage diabetic retinopathy: association between the activation phase of cone phototransduction and the flicker electroretinogram. Invest Ophthalmol Vis Sci (2019) 60:64–72. 10.1167/iovs.18-25946 30640972 PMC6333111

[8] Feigl B Morris CP. The challenge of predicting macular degeneration. Curr Med Res Opin (2011) 27:1745–8. 10.1185/03007995.2011.603301 21777160

[9] Zhang Y Wang Y Shi C Shen M Lu F. Advances in retina imaging as potential biomarkers for early diagnosis of Alzheimer's disease. Transl Neurodegener (2021) 10:6. 10.1186/s40035-021-00230-9 33517891 PMC7849105

[10] Sunness JS Massof RW Johnson MA Bressler NM Bressler SB Fine SL. Diminished foveal sensitivity may predict the development of advanced age-related macular degeneration. Ophthalmology (1989) 96:375–81. 10.1016/s0161-6420(89)32883-1 2710529

[11] Porciatti V. Electrophysiological assessment of retinal ganglion cell function. Exp Eye Res (2015) 141:164–70. 10.1016/j.exer.2015.05.008 25998495 PMC4628896

[12] Scholl HP Zrenner E. Electrophysiology in the investigation of acquired retinal disorders. Surv Ophthalmol (2000) 45:29–47. 10.1016/s0039-6257(00)00125-9 10946080

[13] McAnany JJ Park JC. Temporal frequency abnormalities in early-stage diabetic retinopathy assessed by electroretinography. Invest Ophthalmol Vis Sci (2018) 59:4871–9. 10.1167/iovs.18-25199 30347080 PMC6181244

[14] Ragauskas S Leinonen H Puranen J Ronkko S Nymark S Gurevicius K Early retinal function deficit without prominent morphological changes in the R6/2 mouse model of Huntington's disease. PLoS One (2014) 9:e113317. 10.1371/journal.pone.0113317 25469887 PMC4254453

[15] Asanad S Felix CM Fantini M Harrington MG Sadun AA Karanjia R. Retinal ganglion cell dysfunction in preclinical Alzheimer's disease: an electrophysiologic biomarker signature. Sci Rep (2021) 11:6344. 10.1038/s41598-021-85010-1 33737516 PMC7973731

[16] McAnany JJ Matei N Chen YF Liu K Park JC Shahidi M. Rod pathway and cone pathway retinal dysfunction in the 5xFAD mouse model of Alzheimer's disease. Sci Rep (2021) 11:4824. 10.1038/s41598-021-84318-2 33649406 PMC7921657

[17] Ts'o D Schallek J Kwon Y Kardon R Abramoff M Soliz P. Noninvasive functional imaging of the retina reveals outer retinal and hemodynamic intrinsic optical signal origins. Jpn J Ophthalmol (2009) 53:334–44. 10.1007/s10384-009-0687-2 19763750 PMC5180604

[18] Yao X Wang B. Intrinsic optical signal imaging of retinal physiology: a review. J Biomed Opt (2015) 20:090901. 10.1117/1.jbo.20.9.090901 26405819 PMC4689108

[19] Hanazono G Tsunoda K Kazato Y Tsubota K Tanifuji M. Evaluating neural activity of retinal ganglion cells by flash-evoked intrinsic signal imaging in macaque retina. Invest Ophthalmol Vis Sci (2008) 49:4655–63. 10.1167/iovs.08-1936 18539934

[20] Begum M Joiner DP Ts'o DY. Stimulus-driven retinal intrinsic signal optical imaging in mouse demonstrates a dominant rod-driven component. Invest Ophthalmol Vis Sci (2020) 61:37. 10.1167/iovs.61.8.37 PMC7425724 32721018

[21] Wang B Yao X. *In vivo* intrinsic optical signal imaging of mouse retinas. Proc SPIE Int Soc Opt Eng (2016) 9693:96930H. 10.1117/12.2212810 PMC5289717 28163346

[22] Zhang Q Zhang Y Lu R Li Y Pittler SJ Kraft TW Comparative intrinsic optical signal imaging of wild-type and mutant mouse retinas. Opt Express (2012) 20:7646–54. 10.1364/oe.20.007646 22453443 PMC3387536

[23] Bizheva K Pflug R Hermann B Povazay B Sattmann H Qiu P Optophysiology: depth-resolved probing of retinal physiology with functional ultrahigh-resolution optical coherence tomography. Proc Natl Acad Sci U S A (2006) 103:5066–71. 10.1073/pnas.0506997103 16551749 PMC1405907

[24] Azimipour M Valente D Vienola KV Werner JS Zawadzki RJ Jonnal RS. Optoretinogram: optical measurement of human cone and rod photoreceptor responses to light. Opt Lett (2020) 45:4658–61. 10.1364/ol.398868 32870829 PMC7891461

[25] Pandiyan VP Maloney-Bertelli A Kuchenbecker JA Boyle KC Ling T Chen ZC The optoretinogram reveals the primary steps of phototransduction in the living human eye. Sci Adv (2020) 6:eabc1124. 10.1126/sciadv.abc1124 32917686 PMC9222118

[26] Zhang L Dong R Zawadzki RJ Zhang P. Volumetric data analysis enabled spatially resolved optoretinogram to measure the functional signals in the living retina. J Biophotonics (2022) 15:e202100252. 10.1002/jbio.202100252 34817116 PMC8901551

[27] Jonnal RS. Toward a clinical optoretinogram: a review of noninvasive, optical tests of retinal neural function. Ann Transl Med (2021) 9:1270. 10.21037/atm-20-6440 34532407 PMC8421939

[28] Zhang P Shibata B Peinado G Zawadzki RJ FitzGerald P Pugh EN Jr. Measurement of diurnal variation in rod outer segment length *in vivo* in mice with the OCT optoretinogram. Invest Ophthalmol Vis Sci (2020) 61:9. 10.1167/iovs.61.3.9 PMC7401691 32176260

[29] Lassoued A Zhang F Kurokawa K Liu Y Bernucci MT Crowell JA Cone photoreceptor dysfunction in retinitis pigmentosa revealed by optoretinography. Proc Natl Acad Sci U S A (2021) 118:e2107444118. 10.1073/pnas.2107444118 34795055 PMC8617487

[30] Ma G Son T Kim TH Yao X. Functional optoretinography: concurrent OCT monitoring of intrinsic signal amplitude and phase dynamics in human photoreceptors. Biomed Opt Express (2021) 12:2661–9. 10.1364/boe.423733 34123495 PMC8176815

[31] Cooper RF Brainard DH Morgan JIW. Optoretinography of individual human cone photoreceptors. Opt Express (2020) 28:39326–39. 10.1364/oe.409193 33379485 PMC7771891

[32] Kim TH Wang B Lu Y Son T Yao X. Functional optical coherence tomography enables *in vivo* optoretinography of photoreceptor dysfunction due to retinal degeneration. Biomed Opt Express (2020) 11:5306–20. 10.1364/boe.399334 33014616 PMC7510876

[33] Pandiyan VP Jiang X Maloney-Bertelli A Kuchenbecker JA Sharma U Sabesan R. High-speed adaptive optics line-scan OCT for cellular-resolution optoretinography. Biomed Opt Express (2020) 11:5274–96. 10.1364/boe.399034 33014614 PMC7510866

[34] Berkowitz BA Podolsky RH Childers KL Roche SL Cotter TG Graffice E Rod photoreceptor neuroprotection in dark-reared Pde6brd10 mice. Invest Ophthalmol Vis Sci (2020) 61:14. 10.1167/iovs.61.13.14 PMC7671864 33156341

[35] Deng WT Kolandaivelu S Dinculescu A Li J Zhu P Chiodo VA Cone phosphodiesterase-6γ’ subunit augments cone PDE6 holoenzyme assembly and stability in a mouse model lacking both rod and cone PDE6 catalytic subunits. Front Mol Neurosci (2018) 11:233. 10.3389/fnmol.2018.00233 30038560 PMC6046437

[36] Rosch S Johnen S Muller F Pfarrer C Walter P. Correlations between ERG, OCT, and anatomical findings in the rd10 mouse. J Ophthalmol (2014) 2014:874751–10. 10.1155/2014/874751 24683495 PMC3941775

[37] Wang T Reingruber J Woodruff ML Majumder A Camarena A Artemyev NO The PDE6 mutation in the rd10 retinal degeneration mouse model causes protein mislocalization and instability and promotes cell death through increased ion influx. J Biol Chem (2018) 293:15332–46. 10.1074/jbc.ra118.004459 30126843 PMC6177582

[38] Pennesi ME Michaels KV Magee SS Maricle A Davin SP Garg AK Long-term characterization of retinal degeneration in rd1 and rd10 mice using spectral domain optical coherence tomography. Invest Ophthalmol Vis Sci (2012) 53:4644–56. 10.1167/iovs.12-9611 22562504 PMC3394742

[39] Chang B Hawes N Hurd R Davisson M Nusinowitz S Heckenlively J. Retinal degeneration mutants in the mouse. Vis Res (2002) 42:517–25. 10.1016/s0042-6989(01)00146-8 11853768

[40] Gargini C Terzibasi E Mazzoni F Strettoi E. Retinal organization in the retinal degeneration 10 (rd10) mutant mouse: a morphological and ERG study. J Comp Neurol (2007) 500:222–38. 10.1002/cne.21144 17111372 PMC2590657

[41] Lu Y Kim TH Yao X. Comparative study of wild-type and rd10 mice reveals transient intrinsic optical signal response before phosphodiesterase activation in retinal photoreceptors. Exp Biol Med (Maywood) (2020) 245:360–7. 10.1177/1535370219896284 31852239 PMC7370599

[42] Phillips MJ Otteson DC Sherry DM. Progression of neuronal and synaptic remodeling in the rd10 mouse model of retinitis pigmentosa. J Comp Neurol (2010) 518:2071–89. 10.1002/cne.22322 20394059 PMC2881548

[43] Chang B Hawes NL Pardue MT German AM Hurd RE Davisson MT Two mouse retinal degenerations caused by missense mutations in the β-subunit of rod cGMP phosphodiesterase gene. Vis Res (2007) 47:624–33. 10.1016/j.visres.2006.11.020 17267005 PMC2562796

[44] Yao X Kim TH. Fast intrinsic optical signal correlates with activation phase of phototransduction in retinal photoreceptors. Exp Biol Med (Maywood) (2020) 245:1087–95. 10.1177/1535370220935406 32558598 PMC7400727

[45] Samardzija M Wariwoda H Imsand C Huber P Heynen SR Gubler A Activation of survival pathways in the degenerating retina of rd10 mice. Exp Eye Res (2012) 99:17–26. 10.1016/j.exer.2012.04.004 22546314

[46] Owsley C McGwin G Jr Clark ME Jackson GR Callahan MA Kline LB Delayed rod-mediated dark adaptation is a functional biomarker for incident early age-related macular degeneration. Ophthalmology (2016) 123:344–51. 10.1016/j.ophtha.2015.09.041 26522707 PMC4724453

[47] Hsiao CC Hsu HM Yang CM Yang CH. Correlation of retinal vascular perfusion density with dark adaptation in diabetic retinopathy. Graefes Arch Clin Exp Ophthalmol (2019) 257:1401–10. 10.1007/s00417-019-04321-2 31001668

[48] Litts KM Zhang Y Freund KB Curcio CA. Optical coherence tomography and histology of age-related macular degeneration support mitochondria as reflectivity sources. Retina (2018) 38:445–61. 10.1097/iae.0000000000001946 29210936 PMC6230433

[49] Berkowitz BA Podolsky RH Childers KL Burgoyne T De Rossi G Qian H Functional changes within the rod inner segment ellipsoid in wildtype mice: an optical coherence tomography and electron microscopy study. Invest Ophthalmol Vis Sci (2022) 63:8. 10.1167/iovs.63.8.8 PMC9284466 35816042

[50] Ma G Son T Kim TH Yao X. *In vivo* optoretinography of phototransduction activation and energy metabolism in retinal photoreceptors. J Biophotonics (2021) 14:e202000462. 10.1002/jbio.202000462 33547871 PMC8240094

[51] Kim TH Ding J Yao X. Intrinsic signal optoretinography of dark adaptation kinetics. Sci Rep (2022) 12:2475. 10.1038/s41598-022-06562-4 35169239 PMC8847457

[52] Li Y Fariss RN Qian JW Cohen ED Qian H. Light-induced thickening of photoreceptor outer segment layer detected by ultra-high resolution OCT imaging. Invest Ophthalmol Vis Sci (2016) 57:OCT105–11. 10.1167/iovs.15-18539 27409460 PMC4968769

[53] Mazzoni F Safa H Finnemann SC. Understanding photoreceptor outer segment phagocytosis: use and utility of RPE cells in culture. Exp Eye Res (2014) 126:51–60. 10.1016/j.exer.2014.01.010 24780752 PMC4145030

[54] Kim TH Son T Yao X. Functional OCT angiography reveals early physiological dysfunction of hyaloid vasculature in developing mouse eye. Exp Biol Med (Maywood) (2019) 244:819–23. 10.1177/1535370219850787 31126209 PMC6643197

[55] Chen X Hou P Jin C Zhu W Luo X Shi F Quantitative analysis of retinal layer optical intensities on three-dimensional optical coherence tomography. Invest Opthalmology Vis Sci (2013) 54:6846–51. 10.1167/iovs.13-12062 PMC5963175 24045992

[56] Risseeuw S Bennink E Poirot MG de Jong PA Spiering W Imhof SM A reflectivity measure to quantify Bruch's membrane calcification in patients with pseudoxanthoma elasticum using optical coherence tomography. Translational Vis Sci Tech (2020) 9:34. 10.1167/tvst.9.8.34 PMC7422762 32855880

[57] Meleppat RK Zhang P Ju MJ Manna SK Jian Y Pugh EN Directional optical coherence tomography reveals melanin concentration-dependent scattering properties of retinal pigment epithelium. J Biomed Opt (2019) 24:1–10. 10.1117/1.jbo.24.6.066011 PMC6977406 31254332

[58] Li Y Zhang Y Chen S Vernon G Wong WT Qian H. Light-Dependent OCT structure changes in photoreceptor degenerative rd 10 mouse retina. Invest Ophthalmol Vis Sci (2018) 59:1084–94. 10.1167/iovs.17-23011 29490345 PMC5824802

[59] Bonezzi PJ Stabio ME Renna JM. The development of mid-wavelength photoresponsivity in the mouse retina. Curr Eye Res (2018) 43:666–73. 10.1080/02713683.2018.1433859 29447486 PMC6094161

[60] Volkner M Kurth T Schor J Ebner LJA Bardtke L Kavak C Mouse retinal organoid growth and maintenance in longer-term culture. Front Cel Dev Biol (2021) 9:645704. 10.3389/fcell.2021.645704 PMC8114082 33996806

[61] Linton JD Holzhausen LC Babai N Song H Miyagishima KJ Stearns GW Flow of energy in the outer retina in darkness and in light. Proc Natl Acad Sci U S A (2010) 107:8599–604. 10.1073/pnas.1002471107 20445106 PMC2889335

[62] Bissig D Berkowitz BA. Light-dependent changes in outer retinal water diffusion in rats *in vivo* . Mol Vis (2012) 18:2561. 23129976 PMC3482170

[63] Berkowitz BA Podolsky RH Lins-Childers KM Li Y Qian H. Outer retinal oxidative stress measured *in vivo* using QUEnch-assiSTed (QUEST) OCT. Invest Ophthalmol Vis Sci (2019) 60:1566–70. 10.1167/iovs.18-26164 30995313 PMC6736344

[64] Wang J Saul A Roon P Smith SB. Activation of the molecular chaperone, sigma 1 receptor, preserves cone function in a murine model of inherited retinal degeneration. Proc Natl Acad Sci U S A (2016) 113:E3764–72. 10.1073/pnas.1521749113 27298364 PMC4932934

[65] Trachsel-Moncho L Benlloch-Navarro S Fernandez-Carbonell A Ramirez-Lamelas DT Olivar T Silvestre D Oxidative stress and autophagy-related changes during retinal degeneration and development. Cell Death Dis (2018) 9:812. 10.1038/s41419-018-0855-8 30042417 PMC6057918

[66] Roehlecke C Schumann U Ader M Brunssen C Bramke S Morawietz H Stress reaction in outer segments of photoreceptors after blue light irradiation. PLoS One (2013) 8:e71570. 10.1371/journal.pone.0071570 24039718 PMC3770596

[67] Zhang P Zawadzki RJ Goswami M Nguyen PT Yarov-Yarovoy V Burns ME *In vivo* optophysiology reveals that G-protein activation triggers osmotic swelling and increased light scattering of rod photoreceptors. Proc Natl Acad Sci U S A (2017) 114:E2937–E46. 10.1073/pnas.1620572114 28320964 PMC5389324

[68] Lu CD Lee B Schottenhamml J Maier A Pugh EN Jr Fujimoto JG. Photoreceptor layer thickness changes during dark adaptation observed with ultrahigh-resolution optical coherence tomography. Invest Ophthalmol Vis Sci (2017) 58:4632–43. 10.1167/iovs.17-22171 28898357 PMC5596796

